# Event-Related Brain Potentials for Goal-Related Power Grips

**DOI:** 10.1371/journal.pone.0068501

**Published:** 2013-07-02

**Authors:** Jan Westerholz, Thomas Schack, Dirk Koester

**Affiliations:** 1 Center of Excellence Cognitive Interaction Technology (CITEC), Bielefeld, Germany; 2 Neurocognition and Action Research Group, Faculty of Psychology and Sports Sciences, University of Bielefeld, Bielefeld, Germany; 3 Research Institute for Cognition and Robotics (CoR-Lab), Bielefeld, Germany; University of Plymouth, United Kingdom

## Abstract

Recent research has shown that neurophysiological activation during action planning depends on the orientation to initial or final action goals for precision grips. However, the neural signature for a distinct class of grasping, power grips, is still unknown. The aim of the present study was to differentiate between cerebral activity, by means of event-related potentials (ERPs), and its temporal organization during power grips executed with an emphasis on either the initial or final parts of movement sequences. In a grasp and transportation task, visual cues emphasized either the grip (the immediate goal) or the target location (the final goal). ERPs differed between immediate and final goal-cued conditions, suggesting different means of operation dependent on goal-relatedness. Differences in mean amplitude occurred earlier for power grips than for recently reported precision grips time-locked to grasping over parieto-occipital areas. Time-locked to final object placement, differences occurred within a similar time window for power and precision grips over frontal areas. These results suggest that a parieto-frontal network of activation is of crucial importance for grasp planning and execution. Our results indicate that power grip preparation and execution for goal-related actions are controlled by similar neural mechanisms as have been observed during precision grips, but with a distinct temporal pattern.

## Introduction

The ability to control the movements of our hands is of utmost importance to our daily life. Controlling the hands enables us to perform a wide range of actions, like grasping objects of various shapes, manipulating items, or using tools, all of which involve action transformations. In the middle of the last century, Napier [1] emphasized that the anatomical and biomechanical features of the human hand make it ideal for tool use; grasping can be performed with high precision, but also with strong force. Furthermore, our hands even give us the ability to communicate using gestures, sign language, or writing messages. Because of their clear importance for human action and interaction, manual movements and manual intelligence have become an important topic in cognitive robotics in recent years. Such complex manual movements require anticipatory control, which seems to be based on cognitive networks in long-term memory [2]. Only very few electroencephalographic (EEG) studies have investigated overt complex movements. Up to now, most event-related potential (ERP) studies have either focused on simple movements like button presses or on the preparation phase of a movement. Therefore, we decided to use a grasping task to study the neural mechanisms underlying overt complex human movement control using EEG.

Grasping is a complex and cognitively organized activity. Therefore, it is used in motor control research to investigate the cognitive architecture of goal-oriented action [2]. More than a century ago, Woodworth [3] suggested that goal-directed actions consist of two phases. The first movement phase depends mostly on planning processes that take place before the action. The second movement phase involves discrete feedback-based action control [3], [4]. The anticipatory character of motor planning processes have been demonstrated in a study by Rosenbaum et al. [5], which showed that people chose different initial grips when reaching for the same rod depending on which end they planned to place on a disc on the table. Through this change in initial posture, participants in the study of Rosenbaum and colleagues avoided finishing their movements with awkward end postures (i.e., holding the rod with their thumb pointing down), even if this meant initially grasping the rod with an uncomfortable grip (i.e., an underhand grip). The authors concluded that participants anticipated their future hand postures, as the participants showed a preference for final comfort over initial comfort. This tendency to avoid awkward postures at the final position of a movement was termed the *end-state comfort effect* [5].

Interestingly, such planning processes during a reach and grasp task can be observed on a finer scale than the decision between overhand and underhand grasp. Schütz et al. [6] tested participants in a sequential (predictable) and a randomized (unpredictable) perceptual-motor task, which offered a continuous range of posture solutions for each movement trial. Participants were asked to open a column of drawers in a sequential or randomized order, grasping each drawer on a protruding cylindrical knob. The end-state comfort effect was reproduced under both predictable and unpredictable conditions.

Looking in more detail into the modular organization of grasping, we will find further indicators for anticipation. Before we grasp an object, we reach for it. During this reach phase the fingers preshape in anticipation of the forthcoming grasp. The preshaping of the fingers is not only matched to the object that is grasped, but also to the task that has to be performed with the object [7]. These kinematic effects suggest that anticipation is not only a sensorimotor function, but also a cognitive function reflecting the action goal [7]. In a bar transport task, for example, that replicated the end-state comfort effect, Hughes et al. [8] observed that the formation of the grasp posture started at the beginning of the action. This finding implies that participants had selected a grasp prior to the movement which would satisfy end-state comfort. Moreover, when the action goal was changed shortly after movement onset, participants modified their reach-to-grasp movements to ensure a comfortable posture at the end of the movement, demonstrating the influence of action goals for movement planning and execution.

Different planning processes can, additionally, be observed in the kinematic parameters of power and precision grips [9]. Participants in the study of Castiello et al. [9] had to grasp a small or large dowel and use either a precision grip or a whole hand power grip to do so. On 20% of the trials the object size was unexpectedly changed during the reach phase. The results show shorter movement time and shorter deceleration time for the power grip compared to the precision grip. Maximum grip aperture occurred earlier for the precision grip than for the power grip and, according to Castiello et al. [9], indicates the temporal coordination of grasp and transport components. They suggest that this temporal difference indicates an earlier anticipation of an object’s characteristics in the case of higher precision demands. For trials in which the grip had to be altered during the action, they found changes during the deceleration phase of the reaching movement and, of course, during grasping. Faster movement and deceleration times for power grips indicate that planning processes for these movements must be faster or happen earlier in comparison to the planning processes for precision grips.

There is also neurophysiological evidence for a cognitive function of planning processes toward the action goal, in the form of activation of the motor system during action anticipation [10]. Further neurophysiological studies are likely to discover different variables that influence the spatial and, using EEG, particularly the temporal organization of movement planning and execution.

Following the results of behavioral studies, Majdandzic et al. [11] used fMRI to examine the spatial neuroanatomical organization of movement preparation and the neural correlates of action planning. Their participants inserted an object into one of two slots. The object consisted of a large and a small cube. The two slots matched the objects in size. Participants were given a cue which determined the final goal (which slots to fill) or the immediate goal (which part of the object to grasp). Thus, participants always executed the same movement, but with an emphasis on either of two different parts of the movement sequence. The researchers observed differential preparatory activity along the superior frontal gyrus and in left inferior parietal cortex during the final goal trials, and differential activity in parieto-occipital and occipito-temporal cortex during the immediate goal trials. Their results also show different parieto-frontal circuits responsible for planning of the same action depending on which factors are emphasized. In addition to the previously mentioned study, Castiello and Begliomini [12] report fMRI results that indicate a specific area to be tuned to the type of grasp, namely the anterior intra parietal sulcus. Castiello and Begliomini [12] further suggest that a larger number of precision grip configurations, rather than whole hand grip configurations, might be represented here. Taken together, the aforementioned studies demonstrate the importance of goals for motor control. They suggest that the goals of an action are more crucial for motor planning than the trajectory of the movement itself.

In accordance with the above-mentioned fMRI studies, Filimon [13] found the intra parietal sulcus (IPS) to play an important role for the control of grasping within the distributed parieto-frontal network. Within this network premotor activity seems to precede posterior parietal activity in some instances, depending on the task, parieto-frontal circuit, and effector used. However, the individual contributions of premotor and parietal areas remain unclear. In 2012, Bozzacchi et al. [14] used EEG to investigate temporal aspects of action planning, and they reported some controversial findings. They based their study on the assumption of a parieto-frontal network and recorded pre-movement event-related potentials, more specifically the Bereitschaftspotential (BP). The BP can be observed prior to voluntary movement and is considered to be a manifestation of the preparation for action [15]. One main interest of Bozzacchi et al.’s study was the temporal organization of motor preparation for grasping. Participants performed three different actions: reaching for a teacup, grasping a teacup, and attempting to grasp a teacup while their fingers were constrained by a band, making grasping impossible. Bozzacchi et al. [14] observed activity over parietal areas well before action onset for the goal-oriented action of grasping an object, but not for reaching or impossible grasping. They found that activity for grasping preparation started earlier and was more widespread and complex than was previously described in the literature, as reviewed by Shibasaki and Hallett [16]. Regarding the temporal relation of parietal and frontal activity, Bozzacchi et al. [14] reported that the earliest parietal activity was followed by frontal activity. They conclude that action preparation is affected in an early phase by the meaning of an action as well as by the type of action to be performed.

In a different EEG experiment, Bozzacchi et al. [17] observed similar motor preparation processes for real and virtual grasps (the virtual grasp being a key press, which started a video showing a grasping action) over posterior parietal areas. From this study they conclude that the final action effect, and not the movement kinematics, influenced the early preparation phase. The results provide further support for the suggestion that parietal areas are of crucial importance for grasp planning and that they provide information for grasp preparation. The temporal organization of the neurophysiological correlates underlying grasping and its preparation remains controversial [13]. As far as we know, only few ERP studies have focused on the temporal organization of overt dynamic grasping movements.

Gratton et al. [18] examined the mechanisms of pre- and poststimulus response activation in a choice reaction time paradigm that required an overt movement, namely squeezing a zero-displacement dynamometer. Motor potentials following stimulus presentation suggested that partial analyses of stimulus information could activate responses. Gratton et al. [18] further observed that, at the time of the EMG response, the level of response activation was constant for trials with different response latencies. This study exemplifies that it is possible to investigate the temporal organization of response selection using overt grasping movements.

Van Schie and Bekkering [19] tried to “clarify the individual contributions of the different parts of the motor system that have been implied to underlie goal representations in action control” (p. 184). They instructed a grasp and transport task which dictated either the grasp participants had to use (immediate goal) or the end position of the transport (final goal). Although participants executed the same overt movement in both conditions, Van Schie and Bekkering observed different ERPs for immediate and final action goals. The immediate goal was accompanied by a parieto-occipital slow wave, while the final goal was accompanied by a slow wave over left frontal regions. The authors suggested that the enhanced activation found in posterior parts for the immediate goal indicates this area’s involvement in the prehension of the object. This interpretation is supported by findings of Van Elk et al. [20], who observed enhanced parietal activation for the observation of grip errors and suggested that it reflects a representation of hand-object interaction. The enhanced activation Van Schie and Bekkering found in anterior parts for the final goal might indicate frontal involvement in the planning and control of sequential behavior [19].

In sum, a parieto-frontal network underlying grasping has been shown in several studies. While premotor activity seems to precede posterior parietal activity in some instances [13], Bozzacchi et al. [14] report in their experiment that the earliest parietal activity was followed by frontal activity. Thus, the temporal organization of the neural mechanisms underlying grasping and its preparation remains unclear. The importance of goals for action planning has been shown in behavioral and neurophysiological research. Being able to achieve the goal of an action or performing the same action with an emphasis on either an initial or a final goal all show differences in their respective neurophysiological recordings. These effects suggest different planning processes depending on the specific goals of the action.

Most of these studies addressed neurophysiological activations in precision grasps. In manual action research, the differentiation between power and precision grasps has become increasingly important in the last 20 years for human motor control and cognitive robotics [2]. Power grasps differ in kinematics and cognitive organization from precision grasps. To our knowledge, no previous study has investigated the temporal organization of the brain processes involved in goal-related actions executed with a power grip. Therefore, the aim of the present study is to differentiate cerebral activity and its temporal organization underlying power grips executed with an emphasis on different parts of the action.

In the present study, participants executed a grasp and transportation task with a specified or unspecified power grip. The specified grip condition focused participants’ attention on the initial goal of grasping, while the unspecified grip condition focused their attention toward the final goal of the transport movement. In this regard, our study is similar to the study of Van Schie and Bekkering [19]. Our specified grip condition is comparable to Van Schie and Bekkering’s immediate goal-cued condition, as the participant is given instructions on how to grasp before grasping the cylinder and placing it at the target position. The unspecified grip condition is comparable to their final goal-cued condition, as the participant is given the final location and orientation of the cylinder but no further instruction on how to grasp it. We will use the terms *immediate* and *final goal* hereafter to accentuate the importance of goal-relatedness in our task.

Our hypotheses for the behavioral data are based on the results of Van Schie and Bekkering [19]. Reaction times reflect planning processes before the movement onset [21], [22] and we expect final goal-cued trials to require shorter planning processes compared to immediate goal-cued trials due to the greater congruence with everyday action demands [23], [19]. During reach time, both movement phases of the multiple-process model of limb control [22], which builds on the two-component model of Woodworth [3], overlap. The first phase, which requires planning processes taking place before the action and contains an early corrective component, might be extended for the immediate goal-cued condition compared to the final goal-cued condition due to higher planning demands. As transport times are based on feedback-based control processes and the same movement has to be executed in both conditions, we expect no transport time differences between conditions. We predict that reaction times will be faster for the final-cued condition than for the immediate-cued condition. Reach times might be faster for the final-cued condition in comparison to the immediate-cued condition. We expect no difference for transport times between both cueing conditions.

Given reports of activity in parieto-occipital regions for grasping, and in left frontal regions for reaching the goal of a transport movement [19], we focus specifically on these regions. If it is the case that precision and power grips are processed similarly, we expect to find similar neural mechanisms as those reported by Van Schie and Bekkering [19], which might vary as described below. More specifically, we expect a cueing effect over the parieto-occipital area time-locked to grasping, which is the immediate goal. The activity over parieto-occipital areas for the immediate-cued condition is expected to be more negative overall than the activity for the final-cued condition. Exact time windows for the effects might differ, as the temporal organization of power grips might occur faster in comparison to precision grips. The duration of the deceleration phase of grasping increases with precision requirements [24], [9]. Further, we expect a cueing effect over frontal areas time-locked to movement end, which is the final goal. The activity over frontal areas for the immediate-cued condition is expected to be more positive overall than the activity for the final-cued condition. Van Schie and Bekkering [19] report a significant effect over left and non-significant effect over right anterior prefrontal regions. It has been shown in the past that right-handed participants show larger contralateral activity regardless of the hand used, while left-handed participants show larger contralateral activity only for responses with the left hand [25]. To avoid laterality effects due to differences in handedness, we exclude left-handed participants in this study and counter balanced the side of the executing hand within subjects. Consequently, we expect bilateral ERP effects.

The design of our study allows us to compare cerebral activity for similar movements, that were planned in a different way. The action was planned either with a relative emphasis on selecting a grip (the immediate goal) or with a relative emphasis on selecting a target state (the final goal). Based on the results of Van Schie and Bekkering [19], we predict that the neural processes for action execution, measured by ERPs, will differ between immediate goal-cued and final goal-cued trials. We predict more negative activity for immediate goal-cued trials than for final goal-cued trials over parietal electrodes in the time window from −300 ms to 0 ms time-locked to the immediate goal. As power grips might be processed faster or earlier than precision grips, the predicted negativity might occur earlier as well. Furthermore, we predict more positive activity for immediate goal-cued trials than for final goal-cued trials over frontal electrodes in the time window from −1100 to 0 ms time-locked to the final goal.

## Materials and Methods

### Participants

Eighteen healthy volunteers (mean age 24.39 years; SD 4.06; 13 females) with no known neurological impairments and normal or corrected-to-normal vision participated in the study. All eighteen participants were right-handed, which was evaluated with the use of the Edinburgh Handedness Inventory (mean handedness score: 98,2) [26]. All participants were compensated with course credit or money. The experimental procedure and written consent form for this study were approved by the ethics committee at Bielefeld University, and adhered to the ethical standards of the sixth revision of the Declaration of Helsinki. All participants gave their informed written consent to participate in the study.

### Design and Setup

Participants executed a grasp and transport task under two different conditions. In one condition the action was cued with an emphasis on the immediate goal, and in the other condition the same action was cued with an emphasis on the final goal.

**Figure 1:**
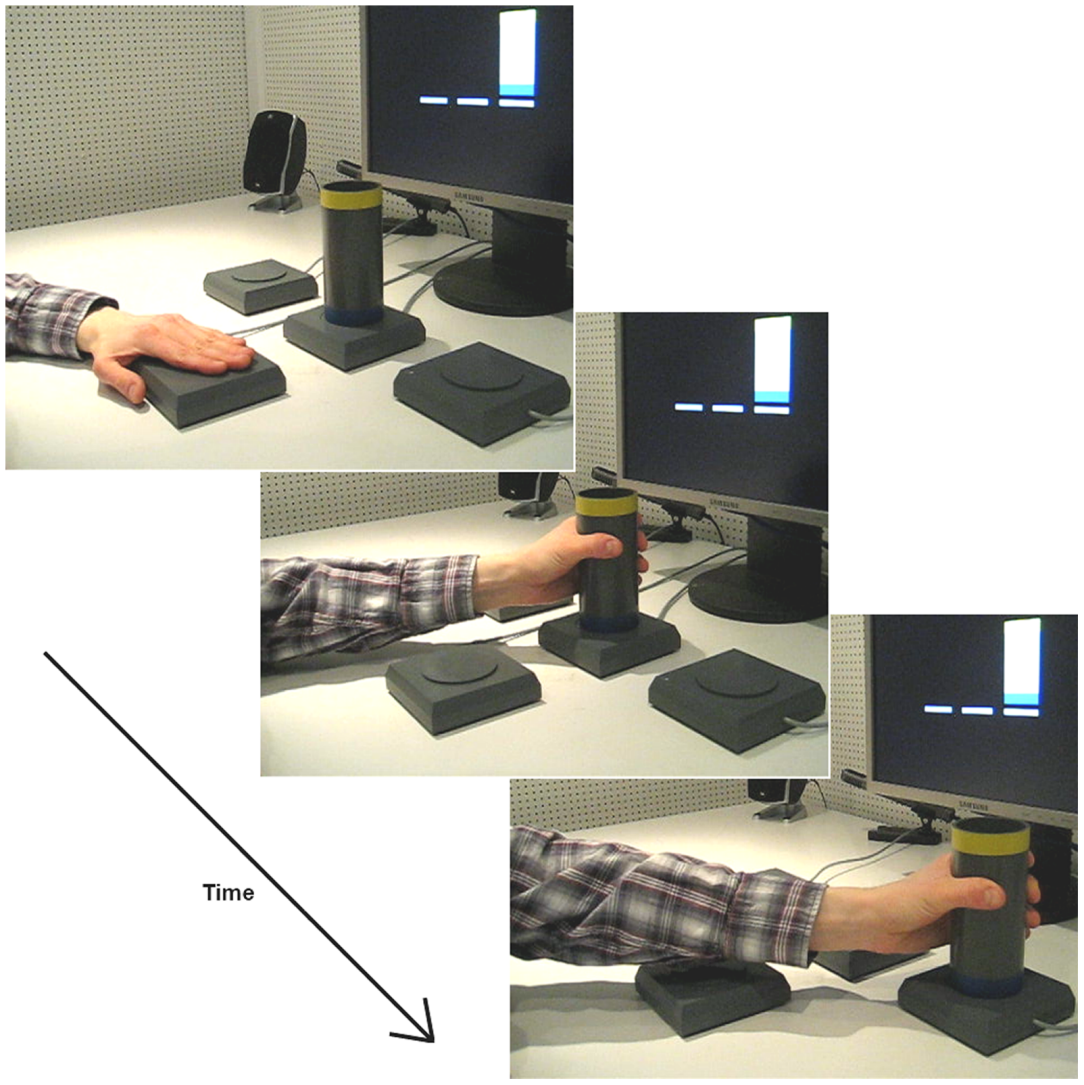
Illustration of the experimental setup. The lateral target locations were aligned shoulder width apart. Both of them could be reached comfortably with an extended arm. The center location and start button were placed directly in the middle in front of the participant. The experiment started with the cylinder on the central location. (TOP LEFT) The participant presses the start button, while the angled arm is resting on the table. A picture stimulus appears on the video monitor. (CENTER) The participant grasps the cylinder. (BOTTOM RIGHT) The participant places down the cylinder on the target location.

Participants were required to grasp an object with a power grip and transport it to a specified goal location. The object was a PVC cylinder with a blue stripe at one end and a yellow stripe at the other end (each about 1 cm in width). The cylinder was positioned on one of three different start/target locations which were aligned next to each other; one on the left, one in the center, and one on the right (see Figure 1). In the center position, the blue mark was always on the bottom side and the yellow mark was always on top.

In each trial, a picture stimulus was presented showing the cylinder in its final location and orientation, which was indicated by the colored marks. The first trial always moved from the center position to either the left or right positions. The next trial was from the lateral location back to the center, bringing the cylinder back to its standard starting position. Only trials from the center to one of the lateral locations entered the analyses.

The cylinder either had to be transported in an upright orientation or it had to be rotated as indicated by the colored marks. At the starting position, the blue mark was at the bottom and the yellow mark was on top. Thus, when the picture stimulus showed the blue mark at the bottom and the yellow mark on top at the final location, the cylinder had to be transported in an upright fashion. Conversely, when the picture stimulus showed the yellow mark at the bottom and the blue mark on top at the final location, the cylinder had to be rotated during transportation. Only trials with an upright orientation of the cylinder during transportation entered the analyses. The other trials served as filler trials, so that participants had to execute different actions and plan their grip anew on every single trial.

Participants performed the task in separated blocks under varying conditions, that is, with different kinds of cues emphasizing different aspects of the action. The first block consisted of picture stimuli showing both colored marks on every trial. Participants grasped the cylinder with a power grip. It was their free decision to grasp with the base of their thumb facing toward the blue or the yellow mark and bring the cylinder to its final location. This cue condition emphasized the final goal. The second block consisted of picture stimuli showing only one of the colored marks. Participants had to grasp the cylinder with the base of their thumb toward the presented mark and bring the cylinder to its final location. This cue condition emphasized the immediate goal. Only trials with the base of the thumb facing upwards in the immediate goal condition entered the analyses. We excluded the trials with the thumb facing down to ensure comparability of the executed movements, because we expected participants to very rarely use this rather uncomfortable grip in the final goal condition. Thus, participants performed the same movement in both blocks, but they were either able to choose the grip themselves or it was pre-specified. The emphasis was either on the immediate goal or on the final goal.

### Procedure

Following electrode preparation, participants were seated comfortably in front of a table in an electrically shielded cabin. Participants received written instruction on the upcoming task. They were given information on how to grasp the cylinder and were instructed to maintain stable posture and not to blink during trials. All questions they had concerning the instructions were answered.

The setup was calibrated to each participant’s size to prevent expansive movements. The lateral locations were aligned shoulder width apart in front of the participants, such that they could reach both of them comfortably with an extended arm. The center location was positioned equidistant to the two lateral locations. The start button was positioned in front of the central location, such that it could be reached with the hand comfortably while the angled arm was resting on the table. Participants were instructed to relax and not to tense up during the action. Picture stimuli were presented on a video monitor located behind the start/target locations. Before the experiment started, participants performed short blocks of test trials to get acquainted to the task. These test blocks were also used to observe the EEG for obvious artifacts and were repeated until participants executed the task correctly in a relaxed state.

**Figure 2:**
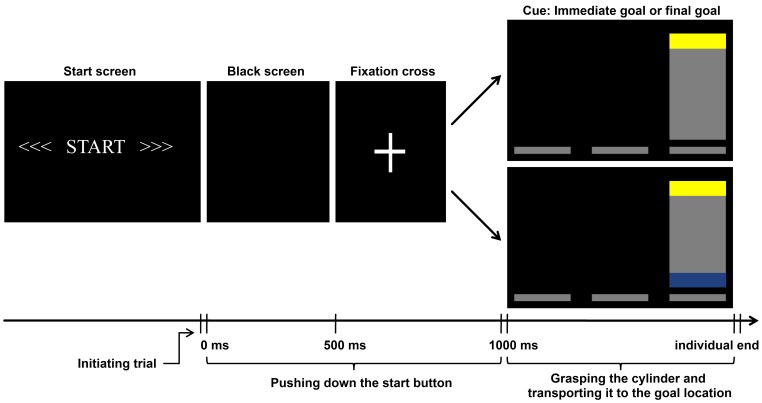
Stimulus sequence for one trial. Time is shown in milliseconds. At the beginning of each trial, the start screen required participants to push the start button. This was followed by a black screen, a fixation cross, and a cue. The cue showed participants to which goal location they had to move the cylinder (only transport to the right is shown). The cue could either emphasize the immediate goal (TOP), or the final goal (BOTTOM), or be a filler trial (not shown). In the immediate goal-cued condition participants had to grasp the cylinder with the base of their thumb towards the color mark shown and transport it to the goal location. In the final goal-cued condition participants had to transport the cylinder to the goal location, the grip was not specified.

Each trial started when participants pressed the start button. First, a black screen was shown for 500 ms, followed by a fixation cross for 500 ms. Next, a picture stimulus was shown indicating the final orientation and location of the object. The stimulus remained on the screen until the end of the trial. Participants then transported the cylinder to the target position (see Figure 2). The timing of all button actions (start, lift off, placing) were registered. Participants repeated each action 40 times (20 with their left hand, 20 with their right hand) for each cueing condition. The stimulus presentation was controlled by Presentation® software (version: 14.1, www.neuro-bs.com).

### Behavioral and Electroencephalographic Recordings

Behavioral recordings included the time points of lifting the hand off the start button, lifting the cylinder, and placing the cylinder down again. Micro switches were used to detect the exact moment they occurred. These events were recorded on the PC which was presenting the stimuli, as well as on the PC which was recording the EEG. Participants’ manual behavior was recorded with a video camera for later offline analysis.

EEG was recorded by a 64 channel amplifier (ANT). A WaveGuard EEG cap (ANT) with sixty-four Ag/AgCl electrodes was used. The electrodes of the cap were arranged according to the international 10–10 system (based on the 10–20 system) [27]. In order to detect ocular artifacts, EOG was recorded using four electrodes placed above and below the right eye and lateral to both eyes. During recording the data were average-referenced. The EEG was band-pass filtered (DC-138 Hz) and digitized at 512 Hz. The impedance of all electrodes was less than 5 kΩ.

### Data Analysis

Video recordings were studied offline for performance errors. A trial was rated as containing an error when the participant used the wrong grip, placed the cylinder on the wrong target, changed the grip during the approach or execution phase of the movement, or dropped the cylinder. Trials with performance errors were excluded from the analyses.

Behavioral analyses for reaction times (time from stimulus presentation to lifting of the hand), reach times (time from lifting the hand to lifting the object), and transport time (time from lifting the object to movement end) were each done separately. Averaged reaction, reach, and transport times were each subjected to a paired t-test to determine the influence of the cue-type (immediate goal-cued, final goal-cued).

Electrophysiological data were band-pass filtered offline from 0.1 to 30 Hz and re-referenced to the average mastoid electrodes. Response-locked analysis to grasping included the time interval from −1500–1000 ms. That means, epochs started 1500 ms before lifting the cylinder from the start position and ended 1000 ms after lifting. Response-locked analysis to movement end included the time interval from −2100–100 ms. That means, epochs started 2100 ms before placing the cylinder down at the target position and ended 100 ms after placing it down. Baseline correction was performed on the first 100 ms of each interval. Ocular artifacts were corrected using the correction procedure of Gratton et al. [28]. Artifact detection was done using a peak-to-peak moving window approach. Epochs containing peak-to-peak amplitudes above the threshold of ±50 µV within a 200 ms window were rejected. This window was moved over the whole epoch in 50 ms steps. Time epochs were visually double-checked for artifacts that would have been missed by the detection algorithm. 20% of the trials time-locked to grasping in the immediate goal-cued condition and 23% in the final goal-cued condition were rejected due to artifacts. 15% of the trials time-locked to movement end in the immediate goal-cued and 17% in the final goal-cued condition were rejected due to artifacts.

The influence of overt movements on EEG recordings is not fully understood yet. However, ERPs have been analyzed successfully and repeatedly in recent studies [29]–[31] which suggests that reliable ERPs can be obtained during overt movements. Importantly, the design of the present study compares conditions in which comparable movements are generated. This means that if there were artifacts still present in the data, these would be the same for all conditions and the reported differences between conditions are highly unlikely to be due to muscle artifacts. Furthermore, the (arm) movements required in our experimental task were comparable to the movements in Van Schie and Bekkering’s study which also supports the expectation of reliable ERP effects for our grasp and transport task.

Mean amplitude analysis of the electrophysiological data included the factors *Cue-type* (immediate goal-cued, final goal-cued), *Front-Back* (anterior, central, posterior) and *Left-Right* (left, middle, right). The ERP was averaged separately for every participant and experimental condition. For the assessment of effects of scalp distribution, we differentiated between nine regions of interest (anterior-left (AL): AF7, F7, F5, F3; anterior-middle (AM): F1, Fz, F2; anterior-right (AR): AF8, F8, F6, F4; central-left (CL): C5, C3, CP5, CP3; central-middle (CM): FCz, Cz, CPz; central-right (CR): C6, C4, CP6, CP4; posterior-left (PL): PO7, PO5, PO3, O1; posterior-middle (PM): Pz, POz, Oz; posterior-right (PR): PO8, PO6, PO4, O2). The Greenhouse-Geisser correction was applied when evaluating effects with more than one degree of freedom (reporting corrected p-values and original degrees of freedom). Note that the EEG data were averaged for the left and right hand responses to avoid handedness effects. Hence, further observed lateral activity should not be evoked by handedness.

We analyzed mean amplitudes of the −300–0 ms time window time-locked to grasping and mean amplitudes of the −1100–0 ms time window time-locked to movement end. In line with the assumption that power grip preparation is faster than precision grip preparation, we also explored the −900 to −500 ms time window time-locked to grasping based on visual inspection.

## Results

Participants executed the task correctly in 96% of trials in the immediate goal-cued condition, and 97% in the final goal-cued condition - the remaining 4% and 3% of trials, respectively, were rejected. We performed a t-test on the arcsine transformed proportions of correct trials. It revealed no significant difference between the immediate goal-cued and final goal-cued conditions, t(17) =  −0.3, p = 0.77.

In the immediate goal-cued condition, 100% of the correct trials were executed holding the cylinder with the thumb up. In the final goal-cued condition, 99.6% of the correct trials were executed holding the cylinder with the thumb up.

### Behavior

We conducted three paired-samples t-tests to compare each of the reaction times, reach times, and transport times in the immediate goal-cued and final goal-cued conditions.

**Figure 3:**
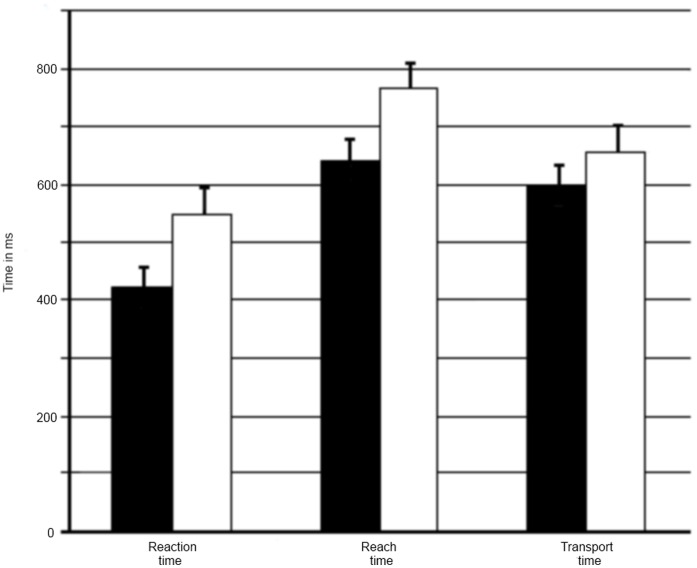
Timing of behavior. Average reaction time, reach time, and transportation time for the final goal-cued condition (black) and the immediate goal-cued condition (grey). The error bars represent standard errors.

Reaction times were faster for final goal-cued trials (422 ms, SD = 148 ms) compared to immediate goal-cued trials (551 ms, SD = 203 ms, t(17) = 4.21, p<0.05)(see Figure 3). According to the multiple-process model of limb control [22], the reaction time can be seen as planning processes happening before movement onset. Thus, the immediate goal-cued condition seems to demand more time to plan the desired action.

Reach times were faster for final goal-cued trials (643 ms, SD = 157 ms) compared to immediate goal-cued trials (767 ms, SD = 198, t(17) = 4.44, p<0.05). Reach time includes both phases of goal-directed aiming as suggested by Elliott et al. [22]. That is, an initial impulse phase containing a corrective component followed by a current control phase. A temporal extension of this phase might point to a longer initial impulse phase, suggesting a more complicated motor plan to be executed; similarly, it could point to a longer current control phase, suggesting online control processes to be more demanding. As the same object has to be grasped and transported in both cueing conditions in our experiment, the online control phase should be of similar difficulty in both conditions. Therefore, this reach time difference suggests that the motor planning processes and possible early corrections of the movement for the immediate goal-cued condition are more complicated than for the final goal-cued condition.

Transport times were faster for final goal-cued trials (602 ms, SD = 150 ms) compared to immediate goal-cued trials (658 ms, SD = 184 ms, t(17) = 2.35, p<0.05). The second phase of the multiple-process model of limb control [22] describes the online control of the movement. This suggests that the transport time might demand more control processes in the immediate goal-cued condition.

In sum, the duration of the whole action sequence was significantly shorter for final goal-cued trials (1667 ms, SD = 329 ms) compared to immediate goal cued trials (1976 ms, SD = 404 ms, t(17) = 4.79, p<0.05).

### Electrophysiology

We conducted an ANOVA time-locked to grasping, which is the moment of lifting the cylinder off of the start position, with the factors Cue-type (immediate goal-cued, final goal-cued), Front-Back (anterior, central, posterior), and Left-Right (left, middle, right). We applied the Greenhouse-Geisser correction when evaluating effects with more than one degree of freedom (reporting corrected p-values and original degrees of freedom).

The ANOVA for −300–0 ms revealed a significant 3-way interaction for Cue-type, Front-Back, and Left-Right, F(4, 68) = 4.51, p<0.05. The 3-way interaction means that the ERP amplitude differences between the immediate and the final goal condition is different in magnitude for the various combinations of the factors Front-Back and Left-Right. The significant interaction permits the separate comparisons of the immediate and the final goal conditions in the various regions-of-interest (ROI). We performed a t-test for every ROI to determine if there was a significant difference based on Cue-type and in which ROI this difference was present. A significant positivity for the immediate goal-cued condition compared to the final goal-cued condition was present in the AR-ROI, t(17) = 2.71, p<0.05. The scalp distribution of the effect in this time window is unexpected and needs to be confirmed by further research. No significant effects were found for the remaining ROIs.

In additional analyses, in line with the assumption that power grip preparation is faster than precision grip preparation, the ANOVA for −900 to −500 ms revealed a significant 3-way interaction for Cue-type, Front-Back, and Left-Right, F(4, 68) = 3.08, p<0.05. Following the 3-way interaction, we performed a t-test for every ROI to determine if there was a significant difference based on Cue-type and in which ROI this difference was present. A significant negativity for the immediate goal-cued condition compared to the final goal-cued condition was present in the PM-ROI, t(17) = −2.14, p<0.05. The negativity was not significant for the PR-ROI, t(17) = −1.97, p = 0.065. A positivity for the immediate goal-cued condition compared to the final goal-cued condition was not significant in the AR-ROI, t(17) = 1.91, p = 0.074. No significant effects were found for the remaining ROIs.

We conducted an equivalent ANOVA time-locked to movement end, which is the moment of placing the cylinder down at the target position, with the factors Cue-type (immediate goal-cued, final goal-cued), Front-Back (anterior, central, posterior), and Left-Right (left, middle, right).

The ANOVA for −1100–0 ms revealed a significant 3-way interaction for Cue-type, Front-Back, and Left-Right, F(4, 68) = 4.3, p<0.05. Following the 3-way interaction, we performed a t-test for every ROI to determine if there was a significant difference based on Cue-type and in which ROI this difference was present. A significant positivity for the immediate goal-cued condition compared to the final goal-cued condition was present in the AR-ROI, t(17) = 2.24, p<0.05. No significant effects were found for the remaining ROIs.

The average duration for the whole action sequence differed between 1667 ms for the final goal-cued condition and 1976 ms for the immediate goal-cued condition. This variability might be associated with the results of the electrophysiological data, because for some trials, especially in the immediate goal-cued condition, the baseline was post stimulus onset, while for most trials it was pre stimulus onset as a consequence of the variable movement times. To rule out an influence of the variability of the time epochs on the observed effects, we conducted further analyses excluding all trials, which included a post-stimulus baseline. The data of participants with less than 10 trials were excluded from further analyses. Data from 15 participants entered analyses response locked to grasping and to movement end. As we narrow down the data based on a temporal factor, the temporal occurrence of the effects might change. To accommodate to these changes and to provide a more detailed account of the temporal domain, we analyzed the data in 100 ms step windows. To correct for false positives we combined these time windows into one, only if three or more consecutive windows revealed significant 3-way interactions for Cue-type, Front-Back, and Left-Right, as well as for according t-tests [32].

In detail, we performed comparable ANOVAs with the factors Cue-type (immediate goal-cued, final goal-cued), Front-Back (anterior, central, posterior), and Left-Right (left, middle, right) for every single 100 ms time window of both epochs (time-locked to grasping and time-locked to movement end). For time windows that revealed a significant 3-way interaction for Cue-type, Front-Back, and Left-Right, we performed t-tests for every ROI. The results of these ANOVAs and t-tests can be found in the supporting information section (Table S1 and Table S2). Only when three or more consecutive intervals reached the significance level (p<0.05), these intervals were combined, that is we averaged the amplitudes, to one time window. As a result, we analyzed in addition the time window from −600 to −200 ms time-locked to grasping and from −700 to −200 ms time-locked to movement end. Thus, the following statistics contain time windows, which consist of series of consecutive 100 ms steps that were found significant.

Time-locked to grasping, the ANOVA for −600 to −200 ms revealed a significant 3-way interaction for Cue-type, Front-Back, and Left-Right, F(4,56) = 3.48, p<0.05. Following the 3-way interaction, we performed a t-test for every ROI to determine if there was a significant difference based on Cue-type and in which ROI this difference was present. A significant negativity for the immediate goal-cued condition compared to the final goal-cued condition was present in the PL-ROIs, t(14) = −2.7, p<0.05, the PM-ROIs, t(14) = −2.86, p<0.05, and the PR-ROIs, t(14) = −2.41, p<0.05. No significant effects were found for the remaining ROIs.

Time-locked to movement end, the ANOVA for −700 to −200 ms revealed a significant 3-way interaction for Cue-type, Front-Back, and Left-Right, F(4,56) = 5.09, p<0.05. Following the 3-way interaction, we performed a t-test for every ROI to determine if there was a significant difference based on Cue-type and in which ROI this difference was present. A significant positivity for the immediate goal-cued condition compared to the final goal-cued condition was present in the AR-ROI, t(14) = 2.36, p<0.05. No significant effects were found for the remaining ROIs.

In sum, the analyses based on the predicted time windows time locked to grasping revealed a right frontal positivity for the immediate goal-cued condition compared to the final goal-cued condition from −300 to 0 ms. They also revealed a parieto-occipital negativity for the immediate goal-cued condition compared to the final goal-cued condition from −900 to −500 ms. The same analyses time-locked to movement end resulted in a right frontal positivity for the immediate goal-cued condition compared to the final goal-cued condition from −1100 to 0 ms. The temporally more fine grained analyses time-locked to grasping revealed a parietal negativity for the immediate goal-cued condition compared to the final goal-cued condition from −600 to −200 ms. Time-locked to movement end, we found a right frontal positivity for the immediate goal-cued condition compared to the final goal-cued condition from −700 to −200 ms.

## Discussion

**Figure 4:**
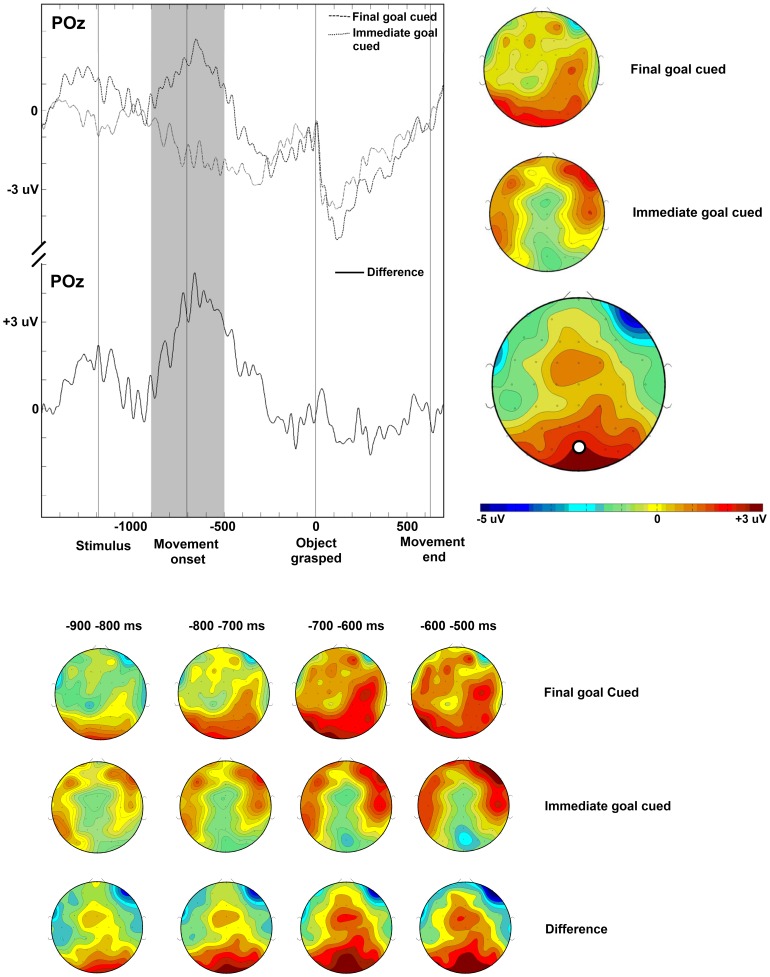
Slow wave brain potentials time-locked to grasping of the object. Time is shown in milliseconds. (TOP LEFT) Event-related slow wave potentials recorded at the medial parieto-occipital electrode POz, time-locked to grasping the object, for the final goal cueing condition (dashed), the immediate goal cueing condition (dotted), and the difference between the two cueing conditions (solid). The labels ‘Stimulus’, ‘Movement onset’, and ‘Movement end’ mark the *average* time points of these events. (TOP RIGHT) Topography of slow waves recorded in the −900 ms to −500 ms time interval before grasping (indicated by the grey selection), in the final goal cueing condition, the immediate goal cueing condition, and the difference between the two cueing conditions. The location of electrode POz on the scalp is illustrated by a white marker. (BOTTOM) Topography of slow waves recorded in the −900 to −500 ms time interval before grasping displayed in consecutive 100 ms intervals, in the final goal-cued condition, the immediate goal-cued condition, and the difference between the two cueing conditions.

**Figure 5:**
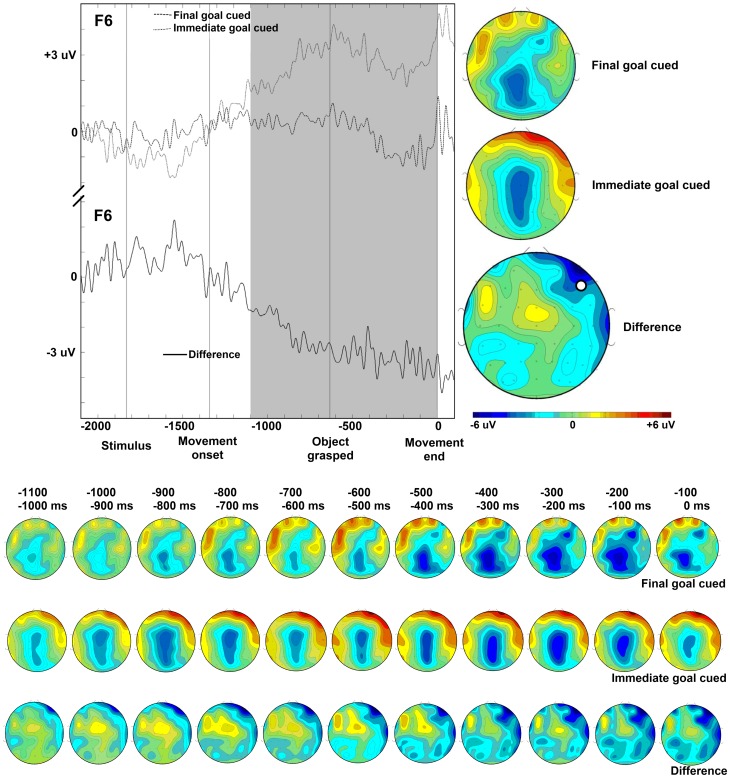
Slow wave brain potentials time-locked to movement end. Time is shown in milliseconds. (TOP LEFT) Event-related slow wave potentials recorded at the right lateral frontal electrode F6, time-locked to movement end, for the final goal-cued condition (dashed), the immediate goal-cued condition (dotted), and the difference between the two cueing conditions (solid). The labels ‘Stimulus’, ‘Movement onset’, and ‘Object grasped’ mark the *average* time points of these events. (TOP RIGHT) Topography of slow waves recorded in the −1100 ms to 0 ms time interval before movement end (indicated by the grey selection), in the final goal cueing condition, the immediate goal cueing condition, and the difference between the two cueing conditions. The location of electrode F6 on the scalp is illustrated by a white marker. (BOTTOM) Topography of slow waves recorded in the −1100 to 0 ms time interval before movement end displayed in consecutive 100 ms intervals, in the final goal-cued condition, the immediate goal-cued condition, and the difference between the two cueing conditions.

This study explored the neurophysiological basis of power grips. More specifically, we studied the functional impact of different goals on the planning and execution of grasping. The aim of the present study was to differentiate cerebral activity for the same action executed with an emphasis on initial vs. final parts of the movement sequence. In a grasp and transportation task, the relative emphasis was either on the grip (the immediate goal) or on the target location (the final goal). As predicted, the neural processes for action execution (measured by ERPs) differed between immediate goal-cued and final goal-cued trials. Time-locked to grasping, we found differential activity between immediate goal-cued and final goal-cued conditions in parieto-occipital regions considerably preceding grasping execution (see Figure 4). We also observed right frontal activity within a time window between −1100 ms and final object placement time-locked to movement end (see Figure 5). These results indicate that power grip preparation and execution for goal-related actions are controlled by similar neural mechanisms as precision grips, but with a distinct temporal pattern.

Participants executed the task correctly in 96% of trials in the immediate goal-cued condition and in 97% of trials in the final goal-cued condition - equally successfully in both cueing conditions. This indicates that task difficulty did not differ between cueing conditions and, hence, that task difficulty is highly unlikely to be related to any effects found between cueing conditions.

As expected, reaction times were faster for the final goal-cued condition. This result is in line with the findings of Van Schie and Bekkering [19], who hypothesized that choosing a movement on the basis of a final goal is a preferred mode of operation. The effect fits also with the position of Rosenbaum et al. [23], who argued that the goals of an action are more critical for action planning than initial hand postures. According to these authors, people prefer to plan actions based on the final goal and not on the immediate goal, like the initial grasp in our experiment.

Reach times, which describe the timeframe from movement onset to grasping, were faster for the final goal-cued condition as well. This might still be attributed to a preferred mode of operation, as it may be unfamiliar for the participants to pick up an object with a prespecified grip in comparison to goal-related grasping. There might be temporal overlap of movement planning with the reach period. It is also possible that planning of the grip during the reach phase affected reach time. If the ‘unfamiliar’ immediate goal-cued condition took more planning effort during reaching, this could have slowed them down. The ‘unfamiliar’ planning might take more effort because participants do not have everyday experience with prespecified grips. Rather, we choose grips in our everyday life based on what we want to do with the object [24].

Another explanation could be that participants were focused on the possibility of making an error in the immediate goal-cued condition. Although the error rate was at a similar level for both cueing conditions, instructions in the immediate goal-cued condition may have focused participants’ attention on the grip and potentially increased their awareness of potential errors in comparison to the final goal-cued condition. In the final goal-cued condition there was no incorrect grip according to the instructions, as it was the participants’ choice which grip to use. In contrast, in the immediate goal-cued condition, the possibility of choosing the wrong grip and consequently making an error existed. With the present data, we cannot decide between these alternative interpretations.

Surprisingly, transport times for the final goal-cued condition were faster than for the immediate goal-cued condition. We did not expect a time difference here because the grip has already been executed and the transport movement is exactly the same. That is, the control phase should not be influenced by processes of grip planning. Again, the difference might be a case of increased awareness of potential errors and participants trying not to make mistakes in the immediate goal-cued condition, and constantly ‘double checking’ their actions. In contrast to our results, Van Schie and Bekkering [19], who did not find a difference for transport times, used a more complicated setup and a more complicated movement had to be executed. A precision grip had to be used to transport an object over a bridge. It is possible that the simpler movement in our experiment caused the effect to spill over from the early movement phase into the next one. This remains speculation until further research has been conducted. Repeating the bar transport task of our experiment with an extension of the movement over a bridge might help to find an explanation.

Consistent with the hypothesis that the neural processes for action execution will differ between immediate and final goal-cued trials, we observed differential activity between the immediate and final goal-cued conditions over parieto-occipital regions for grasping. The differential activity in our study occurred earlier than the activity reported by Van Schie and Bekkering [19], who observed differences between −300 and 0 ms time-locked to grasping. This temporal dissimilarity might be due to the difference in grip type used. It is possible that power grip preparation occurs earlier than precision grip preparation, or does not take as long because power grip preparation is easier. The results of both our analyses show a significant negativity for the immediate goal-cued condition compared to the final goal-cued condition. Temporally, the negativity occurs later in our temporally more fine-grained analyses (−600 to −200 ms), than for the predicted time window (−900 to −500 ms), but it still occurs earlier (300 ms difference in the onset) than the negativity described by Van Schie and Bekkering [19] (−300 to 0 ms). For a long action sequence, like the one we studied, with a temporal variability for execution times among subjects, the neural preparation processes for action execution will vary as well. We narrowed down the data, excluding potentially equivocal trials, for the more fine-grained analyses based on a temporal factor. Thus, the fine-grained analyses might give a more accurate result concerning the timing of the effect. Overall, we see the results of both analyses as a confirmation for the hypothesis that power grip preparation occurs earlier than precision grip preparation, although the exact timing of the process may show some variability. Fewer parameters have to be adjusted for a power grip in comparison to a precision grip. It has already been shown in fMRI experiments that there is increased activity in the anterior intraparietal area (AIP) for increasing precision of the grasp [33], suggesting differences in movement planning between power and precision grasps. This increased neural activity may reflect more effortful planning of precision vs. power grips.

In addition to parietal activity, we observed differential frontal activity between −300 and 0 ms time-locked to grasping, which was not present in the temporally more fine-grained analyses. Van Schie and Bekkering [19] reported frontal activity as well, but only time-locked to movement end. Although it is difficult to compare results time-locked to diverse events per se, it seems that we found a frontal effect in a relatively earlier time window. This variation might also be due to differences between power and precision grips. As the duration of the deceleration phase of grasping increases with precision requirements [24], [9], we can expect the deceleration phase of the whole hand grasp in our experiment to be shorter than the deceleration phase of the precision grip in the experiment of Van Schie and Bekkering [19]. The earlier neurophysiological activity in our study may reflect this different temporal organization of the action.

Frontal activity might follow parietal activity during this grasp and transport task. As the parietal activity occurred earlier in our study, the frontal activity might have started earlier as well. We observed differential frontal activity between immediate and final goal-cued conditions within a time window between −1100 ms and final object placement (i.e., 0 ms) time-locked to movement end. This is in line with the findings of Van Schie and Bekkering [19]. Such an effect can be seen (cf. Fig. 6, p. 189) [19] although they did not report consecutive significant *p*-values for the whole time interval. They reported an anterior left positivity −1100 to −500 ms and −300 to 200 ms for precision grips. Varying from the results of Van Schie and Bekkering [19], who reported significant effects only for differential left frontal activity, we observed differential right frontal activity. The results of both our analyses show this significant positivity for the immediate goal-cued condition compared to the final goal-cued condition. Temporally, the positivity in our more fine-grained analyses, from −700 to −200 ms, lies inside the time interval of the first analyses and in the middle of the time range reported by Van Schie and Bekkering [19]. The exact total duration may differ between the groups of participants. Importantly, the positivity occurs within the wider time window reported by Van Schie and Bekkering [19] which suggests that the functional significance is comparable.

The right frontal activity cannot be explained with the participants’ handedness, as we collapsed data for the left and right hand, i.e., handedness was balanced across participants. An additional visual inspection of each hand’s data suggests that handedness did not influence the present ERP effects. Unfortunately, Van Schie and Bekkering [19] did not explicitly mention whether or not they collapsed data for the left and right hand. Thus, a direct comparison would remain vague.

In sum, we found that ERPs differ between immediate and final goal-cued conditions, suggesting different neural ways of operation dependent on goal-relatedness. The basic pattern of our results was replicated in two analyses. That is, we found an anterior positivity time locked to movement end for the immediate goal-cued compared to the final goal cued-condition and a posterior negativity time locked to grasping for the immediate goal-cued compared to the final goal cued-condition. The posterior negativity appears to occur earlier for power grips than for precision grips, although the exact timing for such a long process varies among participants and needs further confirmation in future research.

Our study confirms the suggestion that parietal areas are of crucial importance in the planning and execution of grasping movements. In accordance with Bozzacchi et al. [14], we observed that parietal activity was followed by frontal activity. They concluded that action preparation is affected by the meaning of an action, precisely by the possibility of executing a desired action. Our results suggest that parietal ERP effects are not exclusively found for the possibility of executing a desired action, but also when planning a power grip with the emphasis directed on different components of the action. Furthermore, we establish these findings for the execution of a power grip, while Bozzacchi et al. [14] focused on the preparation phase of the action. We suggest that action preparation and execution are affected by the goal-relatedness of the action. Our interpretation is also in accordance with Van Schie and Bekkering [19] and confirms the suggestion that immediate and final action goals are supported by different parts of the fronto-parietal network. Again, we establish these findings for the execution of a power grip, while Van Schie and Bekkering [19] focused on precision grips. Precision and power grip preparation and execution seem to be controlled by similar neural mechanisms, but with diverging temporal relations.

Our results for immediate goal-cued and final goal-cued conditions might be seen in parallel to the results of Castiello et al. [9] for precision and power grips. Castiello et al. [9] observed longer movement times for precision grips as compared to power grips, but a relatively earlier time point for maximum grip aperture in precision grips. They argued that this reflects the temporal coordination of grasp and transport components and that this temporal difference might be due to an earlier anticipation of an object’s characteristics in case of higher precision demands. In our case, we observed longer movement times for the immediate goal-cued condition as compared to the final goal-cued condition. We also found a negativity for the immediate goal-cued condition as compared to the final goal-cued condition time-locked to grasping. Van Schie and Bekkering [19] found a comparable effect for precision grips in a later time window. It seems possible that this difference is due to an earlier anticipation [9] of the grasp characteristics in the immediate goal-cued condition compared to the final goal-cued condition, as the cue emphasized the grasping action. We suggest that planning processes can be influenced by manipulating the emphasis on one part of a movement sequence, just like planning processes can be influenced by object characteristics like the size of an object [7], [9].

If we split an action into two phases, as in the two-component model by Woodworth [3] or the multiple-process model of limb control [22], we can see that the two cueing conditions we used in our experiment affected both phases of the action. Both the initial ballistic phase, mainly controlled by planning processes, and the online feedback-controlled phase were affected by the goal cueing condition, as can be seen in the effects for reaction, reach, and transport times, and in the neurophysiological data. The immediate goal-cued condition in comparison to the final goal-cued condition seems to cause more effort for motor planning in both phases of the action. This suggests that executing actions on the basis of the final goal is faster and requires less effort and is thereby the dominant way of planning grasping actions.

We suggest that several components influence grasp planning processes, and the final goal is one of the most influential. Uithol et al. [34] describe dynamic models of hierarchies in motor control. In these models, “elements higher on the hierarchy are represented longer or are more stable than lower ones. As such, they are able to influence an action for a longer time interval, thereby accounting for our capacity to structure behavior around a goal” [34](p. 1083). The effects we found for different goal cueing conditions might be explained within this hierarchy. While the final goal cueing condition might have served as a stable component for the whole action, the immediate goal cueing condition might have been more influential for the first part of the action, until the immediate goal (grasping the cylinder) had been reached.

It might be interesting for future research to investigate the lateralized readiness potential (LRP), which reflects response preparation, in a similar experiment. The present experiment was not designed to maximize hemispheric differences in the electrical signal of motor activity. Therefore, we neither expected, nor reported an effect on the LRP for this experiment. For the present study, we focused on the neural mechanisms underlying grasp planning and execution in relation to the work of Van Schie and Bekkering [19]. In a future study immediate goal-cued and final goal-cued conditions could each be assigned to one hand and within one block, with the assignment of conditions to hands counterbalanced across blocks. A precue could also be used to instruct the hand for the next trial [35]–[37] to randomly mix left and right hand responses within a block. This would enable an investigation of the LRP and, thus, response preparation processes, extending our understanding of the neurophysiological correlates of manual action. In addition, our work also suggests that the combination of ERP recordings with other established experimental grasping designs [38]–[40] can provide valuable insights into the cognitive control of uni- and bi-manual actions.

In conclusion, our results suggest that a parieto-frontal network is of crucial importance for grasp planning and execution. According to our data, parietal activity is followed by frontal activity. To our knowledge, this is the first study to differentiate cerebral activity and its temporal organization underlying power grips executed with an emphasis on different parts of the action. Power grip preparation and execution for goal-related actions seem to be controlled by similar neural mechanisms as precision grips, but with a distinct temporal pattern. Grasp and transport actions seem to be preferably processed in a goal-related manner.

## Supporting Information

Table S1 **100 ms time-step analyses time-locked to grasping.** F-Values for the 3-way interactions of the ANOVAs with the factors Cue-type, Front-Back, and Left-Right; significant values in bold face (p<0.05). ROIs and t-values are reported only for significant effects of Cue-type (immediate goal-cued vs. final goal-cued; p<0.05) as follow-up analyses for significant 3-way interactions; see also text.
(DOCX)

Table S2 **100 ms time-step analyses time-locked to movement end.** F-Values for the 3-way interactions of the ANOVAs with the factors Cue-type, Front-Back, and Left-Right; significant values in bold face (p<0.05). ROIs and t-values are reported only for significant effects of Cue-type (immediate goal-cued vs. final goal-cued; p<0.05) as follow-up analyses for significant 3-way interactions; see also text.
(DOCX)
