# Supplementary material for: Event-Related Brain Potentials for Goal-Related Power Grips
Source: PLoS One. 2013 Jul 2;8(7):e68501. doi: 10.1371/journal.pone.0068501 (PMC3699524; doi:10.1371/journal.pone.0068501)
Supplement: Table S1 — 100 ms time-step analyses time-locked to grasping. F-Values for the 3-way interactions of the ANOVAs with the factors Cue-type, Front-Back, and Left-Right; significant values in bold face (p<0.05). ROIs and t-values are reported only for significant effects of Cue-type (immediate goal-cued vs. final goal-cued; p<0.05) as follow-up analyses for significant 3-way interactions; see also text. (DOCX) [file pone.0068501.s001.docx]

| Time window | -1400  -1300 | -1300  -1200 | -1200  -1100 | -1100  -1000 | -1000  -900 | -900  -800 | -800  -700 | -700  -600 |
| --- | --- | --- | --- | --- | --- | --- | --- | --- |
| F(4,56) | 1.30 | 2.01 | 3.12 | 3.18 | **4.53** | 2.28 | 1.44 | 1.99 |
| t(14) |  |  |  |  |  |  |  |  |
| Time window | -600  -500 | -500  -400 | -400  -300 | -300  -200 | -200  -100 | -100  0 | 0  100 | 100  200 |
| F(4,56) | **3.74** | **3.30** | **3.26** | **3.48** | **3.72** | **4.63** | **4.16** | **3.84** |
| t(14) | -2.66 PL  -2.71 PM  -2.32 PR | -2.72 PL  -2.66 PM  -2.29 PR | -2.73 CM  -2.60 PL  -2.70 PM  -2.37 PR | -2.51 CM  -2.27 PL  -2.61 PM  -2.23 PR |  |  |  |  |
| Time window | 200  300 | 300  400 | 400  500 | 500  600 | 600  700 | 700  800 | 800  900 | 900  1000 |
| F(4,56) | 2.81 | 2.91 | 2.30 | 1.96 | 2.33 | 2.12 | 1.73 | 1.5 |
| t(14) |  |  |  |  |  |  |  |  |
