# Supplementary material for: Event-Related Brain Potentials for Goal-Related Power Grips
Source: PLoS One. 2013 Jul 2;8(7):e68501. doi: 10.1371/journal.pone.0068501 (PMC3699524; doi:10.1371/journal.pone.0068501)
Supplement: Table S2 — 100 ms time-step analyses time-locked to movement end. F-Values for the 3-way interactions of the ANOVAs with the factors Cue-type, Front-Back, and Left-Right; significant values in bold face (p<0.05). ROIs and t-values are reported only for significant effects of Cue-type (immediate goal-cued vs. final goal-cued; p<0.05) as follow-up analyses for significant 3-way interactions; see also text. (DOCX) [file pone.0068501.s002.docx]

| Time window | -2000  -1900 | -1900  -1800 | -1800  -1700 | -1700  -1600 | -1600  -1500 | -1500  -1400 | -1400  -1300 | -1300  -1200 | -1200  -1100 | -1100  -1000 | ...  ... |
| --- | --- | --- | --- | --- | --- | --- | --- | --- | --- | --- | --- |
| F(4,56) | 0.31 | 0.82 | 1.21 | 2.64 | **3.07** | 2.16 | 2.48 | 2.80 | **3.29** | 2.70 |  |
| t(14) |  |  |  |  |  |  |  |  |  |  |  |
| Time window | -1000  -900 | -900  -800 | -800  -700 | -700  -600 | -600  -500 | -500  -400 | -400  -300 | -300  -200 | -200  -100 | -100  0 | 0  100 |
| F(4,56) | 3.21 | **3.66** | **4.04** | **4.88** | **6.29** | **5.57** | **4.45** | **3.84** | **3.43** | **3.18** | **3.41** |
| t(14) |  |  |  | 2.26 AR | 2.47 AR | 2.26 AR | 2.44 AR | 2.28 AR |  |  | 2.20 AR |
